# Factors associated with medical narrative competence of nurses: a qualitative study

**DOI:** 10.3389/frhs.2026.1747696

**Published:** 2026-02-03

**Authors:** Haoyue Zhao, Xiejia Peng, Zongwen Yi, Ping Huang, Xiao He, Yanjia Li

**Affiliations:** Department of Nursing, Sichuan Taikang Hospital, Chengdu, Sichuan, China

**Keywords:** grounded theory, narrative competence, narrative medicine, nurse-patient relationship, nursing communication, qualitative research

## Abstract

**Background:**

The nursing ability for medical narrative is essential for patient-focused practice within the bio-psycho-social model of medicine. This study sought to identify influential factors affecting nursing competence in medical narrative skills among practicing nurses in healthcare settings to determine the personal, environmental, relational, and organizational elements that impact this essential competency using grounded theory and constructivist theoretical frameworks.

**Methods:**

A qualitative study design was employed with semi-structured interviews distributed to nurses with expertise in narrative and communication skills. Twenty nurses were selected through purposive sampling to assess: (1) their narrative medicine knowledge and practice; and (2) factors influencing their narrative competency for those with recognized communication expertise in nursing practice. The study used thematic content analysis for data interpretation. Both individual and contextual data were collected.

**Results:**

Twenty nurses participated in this study. Four major categories of influencing factors emerged: personal factors including lack of narrative medicine knowledge, inadequate communication skills, non-standard behaviors, work dissatisfaction, low self-learning capability, and insufficient technical skills; environmental factors including family life situations, relief benefits, working atmosphere, and hospital humanistic culture; relational factors including patient and family interactions; and organizational factors including problematic training models, low emphasis on narrative skills, and ineffective medical processes. These factors demonstrated complex interrelationships affecting overall narrative competency in nursing practice.

**Conclusions:**

The present findings demonstrate that nursing competence in medical narrative is influenced by complex, interlocking factors at multiple levels. Such evidence can be used to support nursing policy and practice improvement through comprehensive strategies including narrative medicine education, incentive-based compensation systems, adequate staffing and supportive environments, primary nursing care models, and integrated organizational approaches for humanistic nursing practice quality.

## 1 Introduction

The World Health Organization's global health programs and the 2030 Agenda for Sustainable Development have established universal health coverage (UHC) as a founding principle for global healthcare (1, 2). UHC is considered essential for healthcare transformation, involving not only improved access to quality healthcare but also a fundamental shift in healthcare models toward person-centered care that meets patients' comprehensive needs (3). Such healthcare transformation requires improving healthcare systems by focusing on humanistic approaches to care, creating effective partnerships between healthcare providers and patients, and implementing care models addressing patients' physical, mental, and social needs (4). Humanistic care integration has been identified as a fundamental element in achieving healthcare equity and improving healthcare quality globally by providing a complementary bridge between medical competence and patient-centered care delivery (5). Healthcare organizations across the globe now realize that humanistic care implementation is not only a component of legal regulations but also an essential element for achieving competitive advantage and quality improvement in healthcare (6). Within this critical realm, narrative skills have emerged as fundamental competencies for healthcare professionals, serving as an important bridge between clinical practice and humanistic approaches (7).

Narrative medicine was initially conceptualized by Dr. Rita Charon in 2001, who defined it as practicing medicine with narrative competence, highlighting the critical interaction between science and humanities. Subsequently, a more comprehensive definition was developed by an international team of experts in 2014, stating that “narrative medicine is a significant tool for obtaining, understanding, and integrating all facets of various participants in a particular disease experience. This thus ensures humanization and efficiency in healthcare delivery” (8). The core of this competency involves sensing, absorbing, and interpreting others' experiences and responding accordingly (7, 8). Narrative medicine represents an innovative approach to healthcare that bridges technical expertise with humanistic understanding.

In recent years, studies on nurses’ medical narrative ability have received considerable attention, particularly within the Chinese healthcare setting. However, current literature has primarily focused on assessing levels of narrative competency and implementing training programs, with minimal investigation into the factors that affect nurses' narrative abilities (9, 10). Although studies have examined the effects of education on narrative medicine, there remains a substantial gap in understanding the complex and multidimensional variables affecting nurses’ narrative competency (11). Narrative competency has been evaluated extensively through quantitative approaches without adequately exploring the lived experiences and challenges nurses encounter when practicing narrative medicine (12). The interplay among personal, environmental, and organizational factors influencing narrative competency remains understudied (13). Without such comprehensive understanding, effective strategies for improving narrative competency among nurses cannot be developed. In Chinese healthcare environments specifically, where biomedical practices predominate and humanistic healthcare concepts remain underdeveloped, understanding the complex variables influencing narrative competency is essential for developing effective narrative medicine strategies (14). Therefore, an in-depth qualitative exploration is needed to identify the multi-level factors affecting nurses' medical narrative abilities, thereby providing essential insights for developing targeted interventions and creating supportive environments for narrative practice in nursing.

Narrative competence in medicine facilitates humanistic care practices and supports the effective implementation of narrative medicine by medical staff. Consequently, it is essential for all medical staff to develop this competence (15). As a component of humanistic care, narrative competence enables nurses to better comprehend patient experiences with illnesses, reduces emotional distance between nursing staff and patients, and fosters positive transformations in nurse-patient relationships (16, 17). Hospital managers with narrative leadership abilities can effectively promote optimal institutional development through the application of narrative wisdom. Professionals with strong narrative skills are able to build “narrative communities” with patients and their families. These professionals can transcend traditional role identities, participate meaningfully in patient and family stories related to life experiences and illness, rapidly establish empathy with individuals experiencing adverse circumstances, and practice narrative care with compassion. Furthermore, these professionals derive ongoing strength and wisdom for personal and professional growth from patient life stories (12, 18).

This paper examines the multi-level factors influencing nursing professionals' medical narrative competence, offering a novel perspective for nursing education. The objectives of this qualitative research are to identify internal and external factors associated with nurses' medical narrative abilities, explore potentially effective improvement strategies, promote the development and integration of narrative skills, and ultimately enhance nursing's fundamental role in providing quality patient care.

## 2 Methods

### 2.1 Design

This study was grounded in constructivist theory, which posits that individuals construct meaning through their experiences and interactions with the social environment. A qualitative descriptive approach informed by grounded theory principles was employed, as this methodology is particularly suited for exploring the emergence of social processes and developing explanatory concepts (19, 20). Constructivist grounded theory guided the research process through iterative data collection and analysis, constant comparison of emerging concepts, and theoretical sensitivity to participants’ perspectives. Thematic content analysis was applied within this framework to systematically identify patterns emerging from the data while remaining grounded in participants' experiences. Semi-structured interviews were used as the primary data collection method to explore the complex, multidimensional factors influencing nurses’ medical narrative abilities. This approach enabled an in-depth examination of participants’ perspectives and experiences regarding narrative competence in clinical practice.

### 2.2 Setting and sample

This qualitative study was conducted in a Grade A tertiary hospital in China. Participants were selected through purposive sampling. Inclusion criteria required that participants be registered nurses who had been formally recognized or acknowledged for their storytelling or communication skills in clinical practice. Nurses who declined to participate were respected and excluded from the study. Prior to data collection, a member of the research team contacted potential participants via WeChat Enterprise to explain the study's aims and procedures. Nurses who expressed interest and provided consent were enrolled for interviews. Participant characteristics are presented in Table 1.

**Table 1:** General information summary of 20 nurses.

Number	Age (years)	Gender	Education level	Professional title	Working experience (years)	Marital status	Post
YXXSNL001	41	Femininity	Bachelor degree	Supervisor nurse	More than 20 years	Married	Nursing supervisor
YXXSNL002	37	Femininity	Bachelor degree	Supervisor nurse	10–20 years	Married	Nursing supervisor
YXXSNL003	41	Femininity	Bachelor degree	Supervisor nurse	10–20 years	Married	Nursing supervisor
YXXSNL004	42	Femininity	Bachelor degree	Supervisor nurse	More than 20 years	Married	Nursing supervisor
YXXSNL005	43	Femininity	Bachelor degree	Co-chief superintendent nurse	More than 20 years	Married	Nursing supervisor
YXXSNL006	34	Femininity	Bachelor degree	Supervisor nurse	10–20 years	Married	Nursing supervisor
YXXSNL007	39	Femininity	Bachelor degree	Supervisor nurse	10–20 years	Married	Nursing supervisor
YXXSNL008	39	Femininity	Bachelor degree	Supervisor nurse	10–20 years	Married	Nursing supervisor
YXXSNL009	37	Femininity	Bachelor degree	Supervisor nurse	10–20 years	Married	Nursing supervisor
YXXSNL010	36	Femininity	Bachelor degree	Supervisor nurse	10–20 years	Married	Nursing supervisor
YXXSNL011	32	Femininity	Bachelor degree	Supervisor nurse	10–20 years	Married	Nursing supervisor
YXXSNL012	33	Femininity	Bachelor degree	Supervisor nurse	10–20 years	Spinsterhood	Ward sister
YXXSNL013	31	Femininity	Bachelor degree	Supervisor nurse	10–20 years	Married	Ward sister
YXXSNL014	31	Femininity	Bachelor degree	Senior nurse	10–20 years	Married	Ward sister
YXXSNL015	36	Femininity	Bachelor degree	Supervisor nurse	10–20 years	Married	Ward sister
YXXSNL016	33	Femininity	Bachelor degree	Supervisor nurse	10–20 years	Married	Ward sister
YXXSNL017	29	Femininity	Bachelor degree	Senior nurse	4–10 years	Spinsterhood	Ward sister
YXXSNL018	25	Femininity	Bachelor degree	Nurse	1–3 years	Spinsterhood	Ward sister
YXXSNL019	37	Femininity	Bachelor degree	Supervisor nurse	10–20 years	Married	Ward sister
YXXSNL020	31	Femininity	Bachelor degree	Supervisor nurse	10–20 years	Spinsterhood	Ward sister

### 2.3 Data collection

A semi-structured interview guide was developed based on a comprehensive review of relevant literature (21). Prior to formal data collection, a team meeting was conducted to review the interview guide and incorporate feedback from research team members. The guide was subsequently pilot-tested with two participants, and refinements were made based on this preliminary testing. Interviews were conducted either in a private, quiet location or via telephone, according to participant preference and convenience. Written informed consent was obtained from all participants prior to their interviews. The interviewer used flexible, open-ended questioning techniques, with follow-up probes as needed to explore participants' responses in depth. Each interview lasted approximately 30–60 min, and all sessions were audio-recorded to ensure accurate data capture. Data collection was conducted between July and August 2024. The interview guide is provided in Table 2.

**Table 2:** Structured interview guide for evaluating nurses’ medical narrative ability.

Structured interview guide for evaluating nurses’ medical narrative ability
1. Based on your work experience, what do you think is a nurse's medical narrative ability or narrative nursing ability?
2. What do you think of nurses’ medical storytelling ability? Do you think it is important? You can expand on your ideas.
3. Have you ever studied narrative nursing or narrative medicine? Can you talk about your thoughts?
4. How do you feel about your communication skills? How do you usually get along with your patients? What are your experiences and skills in patient communication?
5. Are you satisfied with your current nursing work? Where is satisfaction or dissatisfaction intended? Does it indirectly affect narrative ability?
6. What do you think is the relationship between nurses’ medical narrative ability and humanistic hospital construction?
7. Do you feel that narrative nursing or humanistic nursing is currently practiced in your department? What specific activities have been carried out?
8. In your career as a nurse, what is the most impressive thing you have had with patients?
9. Do you think that the environment and culture of the hospital and the relationship between the medical staff will affect the communication between you and the patients? Can you give a simple example?
10. Are you satisfied with your present salary? How do you think the salary and benefits of nursing staff are related to the ability of medical narrative?
11. Do you reflect on your shortcomings in your work (including communication with patients)? Have you ever thought about how to improve it?
12. How do you feel about your ability to empathize? Do you think from the perspective of patients in your work?
13. Do you have any experience or experience of visiting or accompanying patients in other hospitals? Choose a description of your experience that impresses you most?
14. Do you usually read or write yourself? To record the feelings or reflections on the work (including circle of friends, memos, diaries, etc.)?
15. Do you think the professional competence of the nursing staff is more important or the medical narrative ability is more important?
16. How do you think personal living environment, family relationship and interpersonal relationship are related to nurses’ medical narrative ability?
17. What other aspects do you think affect nurses’ medical narrative ability?

### 2.4 Data analysis

Data was analyzed by means of thematic content analysis with a view to closely scrutinizing gathered data (13, 22). Data was gathered and analyzed concurrently, following an iterative process where each interview was transcribed and preliminarily coded before subsequent interviews were conducted. This concurrent approach enabled the research team to monitor for data saturation throughout the data collection process.

Data saturation was operationally defined as the point at which no new themes, codes, or meaningful variations emerged from additional interviews (23). A stopping rule was employed whereby data collection would continue until at least three consecutive interviews yielded no new codes or themes. During analysis, thematic saturation was observed after the 17th interview, when no new major themes or subcategories emerged. Interviews 18 through 20 were conducted to confirm saturation, and these final three interviews yielded no additional codes or themes, confirming that adequate data saturation had been achieved.

Two independent researchers routinely scrutinized transcribed data to gain a comprehensive understanding. Key terms related to similar concepts were identified and coded by both researchers. These formed textual explanations, which were collated into subcategories and categories and finally compiled into overarching themes. After rigorous summarization and thematic synthesis, clear themes developed. The process of data gathering allowed for refinement of these themes. The first author developed themes, which were verified by other investigators after scrutinizing both transcriptions and recordings. All authors engaged in multiple discussions and finally arrived at consensus on overarching themes.

### 2.5 Rigor

Methodological rigor was established through Lincoln and Guba's four criteria: credibility, confirmability, transferability, and dependability (24). Credibility was enhanced through prolonged engagement with participants, semi-structured interviews that allowed for in-depth exploration of experiences, and researcher triangulation during data analysis. Confirmability was achieved through independent review of transcripts by two researchers and maintenance of an audit trail documenting analytical decisions. Transferability was supported by providing thick descriptions of the study context, participant characteristics (including age, educational background, and work experience), and the research setting, enabling readers to assess the applicability of findings to other contexts (24). Dependability was ensured through clear documentation of the research process, including data collection and analysis procedures, enabling future replication of the study methods.

### 2.6 Ethical considerations

This study was approved by the Institutional Review Board of Sichuan Taikang Hospital (No. SCTKIRB-2024-014) and was conducted in accordance with established ethical guidelines for human subjects research. All participants received a detailed explanation of the study procedures and provided written informed consent prior to participation. Participant confidentiality was maintained throughout the study by assigning identification codes and storing data securely. Personal identifying information was removed from all transcripts and research reports.

### 2.7 Sample description

A total of 20 female nurses were successfully recruited, with no refusals or dropouts. The mean age of participants was 35.2 years (range: 25–45 years), and all held bachelor's degrees (Table 1). Regarding professional positions, the sample included 1 staff nurse, 2 nurse practitioners, 16 senior nurse practitioners, and 1 associate director of nursing. With respect to clinical experience, 1 participant had 1–3 years, 1 had 4–10 years, 15 had 10–20 years, and 3 had more than 20 years of experience. All participants were employed at the same Grade A tertiary hospital.

Analysis of the interview data revealed four major thematic categories: personal factors, environmental factors, relational factors, and organizational factors. Each theme encompassed multiple subcategories, which are presented below with supporting quotations from participants.

## 3 Results

Four major thematic categories emerged from the analysis of interview data: personal factors, environmental factors, relational factors, and organizational factors. Each category comprised multiple subcategories that collectively influenced nurses' medical narrative abilities. The findings are presented below with representative quotations from participants.

### 3.1 Personal factors of nurses

#### 3.1.1 Insufficient knowledge of narrative medicine

Participants reported a lack of systematic training on narrative medicine for nurses. This absence may contribute to nursing staff focusing primarily on patient physiological monitoring and treatment implementation, potentially limiting their attention to patients' emotional, psychological, and social needs. Three participants (L1, L9, L19) acknowledged that their familiarity with narrative medicine was shallow, explaining that their understanding was limited to general concepts of providing high-quality or humanistic care. Similarly, the majority of other participants (L3, L4, L6-L8, L11, L14, L18, L20) reported that they had not received systematic training in this area and possessed only superficial knowledge. This inadequate understanding of narrative medicine may impede nurses from improving patient care and potentially result in insufficient emotional support and understanding for patients.

#### 3.1.2 Lack of narrative nursing communication skills

Effective narrative medicine practice requires nurses to possess strong communication skills that enable them to engage patients in sharing experiences and facilitate active listening. However, communication skills among some nurses were identified as a barrier to these discussions and to obtaining the information required for individualized, patient-centered care. Four participants acknowledged that their communication skills were inadequate, describing their patient communication as reactive rather than proactive—responding to patient queries rather than initiating in-depth dialogue:

For instance: (tipping head slightly) Communication skills are not strong. Thorough interaction with patients is required (L5).

Simply greeting and asking “What can be done to help you?” (L4).

Several other participants described their communication skills as moderate, noting that these skills varied depending on circumstances during ward rounds or patient assessments. These nurses attempted to address communication needs expressed by their patients:

Ward rounds or inspection activities are dependent on what patients would like to communicate (L14, L16, L17).

#### 3.1.3 Variations in professional behavioral standards

Professional behaviors such as respect, care, and empathy are considered essential criteria for nursing professionalism. Participants identified variations in adherence to these behavioral standards—including inconsistent displays of warmth, patience, and attentiveness—as factors affecting narrative engagement. Four participants emphasized that maintaining a positive demeanor, demonstrating affinity, and using a gentle approach are essential for facilitating harmonious relationships between healthcare providers and patients, which in turn enhances patient comfort and fosters narrative engagement:

Examples: Attentive listening, a pleasant personality, and a high affinity have been found to enhance patient experience (L7).

Two participants highlighted that patience, effective communication skills, and respect for patient privacy are also critical for establishing a trusting atmosphere and laying the groundwork for practicing narrative medicine:

Irregular responses or sharing patient information greatly worries patients (L9).

#### 3.1.4 Dissatisfaction with work status

Work dissatisfaction among nurses was identified as a threat to the practice of narrative medicine. Three participants reported challenges in displaying patience and empathy in communication due to diminished feelings of worth, passion, and motivation, compounded by pressure resulting from hospital departmental restructuring. Consequently, these nurses often neglected patient narratives:

For instance, no value, passion, motivation, or courage to communicate with the patient and the family members (L5).

Three additional participants described heavy workloads and pressure from interdisciplinary collaboration and departmental coordination, which left them with insufficient time for in-depth patient communication and impaired their listening abilities:

Shortcomings are found and attended to every day, with no time for communication with the patient (L8). This constant focus on identifying and correcting deficiencies left little opportunity for meaningful narrative engagement with patients.

Besides regular duties, one should be on call 24 h a day (L15).

These negative emotions and workload pressures were found to hinder the development of nurse-patient relationships built on trust and empathy, thereby negatively impacting narrative medicine practice.

#### 3.1.5 Lack of self-learning ability

Participants indicated that many nurses lack self-directed learning skills in narrative medicine and tend to rely heavily on external instruction, often employing ineffective learning strategies. This dependency makes it difficult for nurses to independently develop, organize, and apply relevant knowledge in practice, thereby impeding timely enhancement of their narrative competencies. Several respondents reported that reliance solely on external teaching without personal initiative hinders the internalization of concepts and effective skill development.

For instance, one participant stated: “They do not have motivation for self-directed learning; they just depend on external training, which makes it hard to improve abilities” (L1).

#### 3.1.6 Insufficient professional technical skills

Insufficient professional technical skills were identified as a barrier to nurses practicing narrative medicine. Two participants noted that possessing narrative skills alone, without solid professional nursing skills, is ineffective for gaining patients' confidence:

“Narrative skills by themselves will not build confidence with patients. Without nursing skills, it will be hard. Without nursing skills, words alone have little value” (L1).

Three additional participants emphasized that professionalism serves as the foundation for establishing trusting healthcare provider-patient relationships, as patients primarily seek clinical expertise:

Competence is basic. Clients visit healthcare facilities for the sole aim of solving their medical problems through expertise (L5).

With such high volumes, it is necessary to focus on professionalism and competency, with any spare time used for humanism and story (L20).

These findings suggest that professional skill development is a critical component of successful narrative medicine practice.

### 3.2 Surrounding environmental factors

#### 3.2.1 Individual family living environment

Family was identified as a significant source of emotional support for nurses. Participants indicated that a warm and harmonious family atmosphere is essential for maintaining a positive mindset. Supportive family circumstances enable nurses to understand and care for patients more effectively, thereby enhancing their medical narrative abilities. Conversely, economic difficulties and family conflicts can adversely affect nurses' emotional states and work performance. The majority of participants agreed that individual personality is shaped by family atmosphere, with diverse backgrounds and education levels contributing to varying perspectives:

For Instance: Various life and family environments evoke varied personality types (L1).

A harmonious family atmosphere and good family relationships boost nurses' moods and thus improve interactions with patients (L8, L15).

These findings indicate that a supportive family environment plays a significant role in influencing nurses' narrative abilities, as emotional well-being is essential for delivering quality nursing care.

#### 3.2.2 Welfare benefits

Attractive compensation and benefits packages were reported to contribute to job satisfaction among healthcare providers. Participants suggested that adequate compensation may help reduce financial concerns, thereby creating more favorable conditions for engaging in patient-centered activities such as narrative practice.

For example: “Salary is a measure of one's strength and capabilities; one's competency should be rewarded for improvement (L10).”

Narrative skills require investment of time and personal interest. Hence, these skills should be rewarded in terms of salary for their value in nursing (L17).

Higher pay will encourage nurses to work harder and more diligently (L20).

#### 3.2.3 Departmental work atmosphere

A positive work environment was found to enhance the motivation of healthcare staff and increase their engagement in patient care activities. Supportive departmental environments were reported to foster collaboration among team members:

The environment matters. If my co-workers improve for the better, then I improve for the better (L8).

It is necessary that synchronization between doctors and nurses is ensured, with a relaxed atmosphere (L9, L4).

If top organizational leaders exude humanistic care, it would allow nurses to empathize with patients more easily (L11).

#### 3.2.4 Hospital humanistic environment

A hospital environment characterized by humanistic values was found to promote patient respect and compassionate care, while fostering empathy among nursing staff. In such environments, nurses are better positioned to understand patient needs by adopting patients' perspectives, thereby developing a sense of responsibility and attentiveness toward patient stories:

Narrative skills must extend even farther than theory to practice. Hospital cultural environment, humanistic publics, convenient strategies, signage, and experiences with visiting all play a part (L2).

An appropriate hospital setting can alleviate emotional stress, increase confidence during communication, and promote communication with patients. Therefore, narrations can be encouraged for implementation (L4, L7, L16, L19).

### 3.3 Nurse-patient relationship factors

#### 3.3.1 Relationship with patients

Participants reported that when healthcare professionals express genuine interest in patients' personal experiences and feelings, this contributes to increased patient trust and encourages patients to disclose more information. Active listening by healthcare professionals enables nurses to comprehend patient needs, develop individualized care plans based on patient preferences, and ultimately improve patient outcomes:

Trust leads to pleasant relationships (L 1, 9, 11, 13, 15).

They might question their treatment, but communication can foster their trust (L3).

Earlier, one patient refused cesarean delivery. After careful explanations, with emphasis on safety for the baby, both mother and child are now safe (L9).

#### 3.3.2 Relationship with patients' families

Participants recognized that family members often possess valuable knowledge about patients’ situations and circumstances. Effective communication with family members was found to help nurses develop a comprehensive understanding of patients. Additionally, the emotional states of family members can significantly impact patients, and building positive relationships with family members can provide essential emotional support:

For instance: Without coming to a consensus and understanding family intentions, it is easy for conflicts to arise (L2).

Earlier, one patient was not cooperative, and the family members were very nervous. After explanations about the disease, the family members cooperated actively with the patient. Later, even during a leadership inspection, they repeated information that had been explained earlier (L8).

An example would be a child with jaundice. The family was initially reluctant to transfer to pediatrics. Questions were posed, which required careful explanations. Once this was done, treatment followed, and the family was appreciative (L9). These examples illustrate how effective family communication enables nurses to understand the broader narrative context surrounding patient care and to incorporate family perspectives into their narrative practice.

### 3.4 Institutional factors of medical institutions

#### 3.4.1 Lack of diversified training and education models

Participants identified a need for more diverse and interactive training approaches for developing narrative competence. They suggested that experiential learning methods could enhance engagement and learning outcomes:

Presenting patient experiences, group discussions with workshops, and role-play activities greatly increase nurses' engagement and learning achievements (L1).

Playing out a scenario by involving real patients and allowing trainees to assess interactions with questioning encourages interest and critical thinking (L5).

Participants showing work examples, sharing experiences, and involving others in analytical discussions facilitate learning (L10).

Role reversal enables trainees to have firsthand experiences in caregiving, thus improving their grasp of narrative care (L16).

#### 3.4.2 Lack of emphasis on nurses’ medical narrative ability

Participants reported that healthcare institutions and nursing education programs do not place adequate emphasis on developing nurses' narrative competency. This lack of organizational priority makes it challenging for narrative medicine concepts to be disseminated effectively and for nursing staff to fully understand and appreciate the values of narrative medicine and nursing.

For example:

Indeed, to grasp the concept of narrative medicine and narrative nursing properly means recognizing advantages such as enhancing service quality and caregiving ability (L2).

It is necessary for all individuals, including non-clinical staff, to be aware and appreciate the importance of narrative medicine (L5, L13).

#### 3.4.3 Need to optimize the medical treatment processes in institutions

Participants identified that lengthy administrative procedures consume the time and energy of healthcare providers, making it difficult for them to genuinely listen to patients' illness experiences. These inefficiencies increase patient dissatisfaction, further hindering effective communication between healthcare professionals and patients:

Also, there are issues with regards to the hospital's medical treatment process, which is dealing with a large volume of patients (L13).

The outpatient facilities should enable patients to visit easily (L3). It is because of issues related to outpatient activities and hospital structures that nurses can only do their best in coordinating, communicating, and optimizing (L6). Streamlined processes would afford nurses more time to engage in meaningful narrative interactions, allowing them to listen to and respond to patients' illness stories rather than focusing primarily on administrative tasks.

## 4 Discussion

This qualitative study provided a comprehensive exploration of the multiple dimensions influencing narrative medicine competency among nursing staff in a Chinese healthcare setting. The findings revealed that personal, environmental, relational, and organizational factors interact in complex ways to affect nurses' narrative medicine competency, providing critical insights for developing practice improvement strategies (10, 25). The identification of these four distinct categories underscores the importance of adopting a systems-oriented perspective when seeking to enhance narrative competency among nursing staff (26). These comprehensive findings have significant implications for healthcare systems worldwide that seek to integrate humanistic practices with clinical professionalism, as they highlight the multifaceted nature of developing narrative competence within healthcare organizations (7).

The findings indicated that personal factors significantly influence nurses' narrative competency. Specifically, limited formal education on narrative medicine concepts and insufficient training in therapeutic communication techniques were identified as key barriers. Most participants possessed only superficial awareness of narrative medicine concepts, frequently conflating them with general quality patient care or humanistic nursing practice. This finding is consistent with prior research reporting that knowledge deficits represent a primary barrier to implementing narrative practices (27, 28). This gap is particularly concerning because narrative competence involves complex skills such as recognizing, absorbing, interpreting, and responding to patient stories (29). According to narrative medicine theory, competence encompasses both theoretical understanding and practical skills, yet participants demonstrated inadequacies in both domains (30). Additionally, nurses' work dissatisfaction and limited self-learning abilities further impeded the development of narrative competence (31).

These findings suggest that healthcare organizations should develop comprehensive educational programs on narrative medicine that integrate both theory and practice for nurses (32). It is recommended that healthcare organizations incorporate systematic narrative medicine training into continuing nursing education programs, including reflective narrative writing workshops, case discussions, and supervised practice activities (33). These recommendations align with the objectives of “Healthy China 2030,” which emphasizes enhancing healthcare professionals' skills for patient-centered care and improving healthcare quality (34). Furthermore, it is suggested that nursing educational programs incorporate narrative medicine concepts throughout the curriculum, including advanced practice programs, to facilitate cumulative skill development among nurses (35).

Environmental factors, including family environment, compensation and benefits, departmental working atmosphere, and hospital humanistic culture, were found to play a significant role in nurses' narrative practice skills (36). Nurses with harmonious family situations and supportive working environments demonstrated higher levels of emotional receptivity to patient stories, a finding that supports prior literature suggesting that workplace well-being is associated with compassionate caregiving (37). The substantial influence of compensation and benefits on narrative practice indicates that humanistic practice may be constrained by material circumstances, as economic hardship may diminish nurses' capacity for compassionate caregiving (38). This observation is consistent with Maslow's Hierarchy of Needs theory, which posits that basic physiological and security needs must be fulfilled before individuals can engage in higher-order caregiving behaviors. Furthermore, healthcare organizations with humanistic working cultures were found to positively influence nurses' commitment to narrative practice (39).

Based on these findings, healthcare organizations should prioritize creating “narrative-friendly” environments through multiple strategies (40). These include developing competitive remuneration packages that recognize narrative skills, fostering team environments that promote collaboration and value reflective practices, and designing physical spaces conducive to private discussions between patients and nursing staff (41). These recommendations are consistent with the “Further Improvement of Nursing Services Action Plan (2023–2025),” which emphasizes improving nursing service quality and patient satisfaction through capacity building, quality enhancement, and sustainable development. Additionally, narrative medicine peer support groups and communities of practice should be established to enable nurses to share experiences and collectively develop narrative competence (42).

The quality of nurse-patient relationships emerged as a critical factor influencing nurses' engagement in narrative practice (43). Participants described how mutual trust facilitated deeper narrative interactions, while time constraints and communication barriers hindered relationship development. This finding is supported by Peplau's theory of interpersonal relationships, which positions the nurse-patient relationship as central to therapeutic nursing practice (44). Notably, the emergence of nurse-family relationships as an additional essential element expands upon prior research, which has tended to focus primarily on dyadic nurse-patient interactions in narrative practice. Participants reported that positive relationships with patients' families enhanced nurses' understanding of patients' life circumstances, thereby facilitating comprehensive narrative practice. Recent studies have demonstrated that nurses' narrative abilities significantly influence communication effectiveness with challenging patients through reflective practice, which in turn strengthens therapeutic relationships. However, participants also noted that managing complex family dynamics involving conflicting narratives presented challenges for which they felt inadequately prepared.

To address these relational factors, healthcare organizations should consider implementing structured approaches to relationship development. Such approaches may include adopting primary nursing models that facilitate long-term nurse-patient relationships, allocating dedicated time for narrative interactions, and providing training focused on active listening skills. The incorporation of family-centered care principles, as advocated in contemporary nursing initiatives, can support the development of comprehensive narrative practices that honor all stakeholders' stories. Furthermore, healthcare organizations should recognize and reward excellence in relationship-based nursing care through formal performance evaluation systems.

The organizational factors identified in this study, including limitations in training approaches, insufficient organizational emphasis on narrative skills, and inefficient healthcare processes, represent significant barriers to implementing narrative practices (39). Participants consistently reported that traditional training models failed to develop practical narrative skills, while organizational priorities favored technical tasks over relational care (40). These findings are consistent with international literature indicating that institutional culture and structures play a critical role in determining the feasibility of humanistic care practices (41). Specifically, the reliance on traditional didactic teaching methods was identified as inadequate for developing the complex skills required for narrative practice (42). Recent studies have demonstrated that nursing organizational culture mediates the relationship between leadership styles and nurses' clinical competencies, suggesting that transforming institutional culture is essential for advancing nursing practices such as narrative competence (45). Additionally, inefficient healthcare processes were found to deplete resources that could otherwise support narrative engagement.

Addressing these organizational barriers requires comprehensive institutional change. Healthcare organizations should revise their education programs to include experiential learning approaches such as narrative medicine rounds, reflective practice groups, and guided storytelling sessions. Process improvement initiatives should aim to reduce administrative burdens on healthcare staff, thereby maximizing opportunities for patient engagement. The designation of narrative medicine champions or coordinators within nursing departments can help maintain momentum for narrative practice development across organizations. These recommendations align with current healthcare strategies promoting patient-centered care delivery, quality improvement, and the integration of humanistic approaches into clinical practice. Furthermore, organizations should incorporate narrative competence criteria into performance evaluation systems and allocate dedicated financial resources to narrative medicine programs, thereby demonstrating institutional commitment to this essential nursing competency.

Based on these findings, nursing managers are encouraged to initiate comprehensive strategies addressing these multifaceted factors. Recommended strategies include: integrating systematic narrative medicine education into continuing education programs, establishing mentoring initiatives, and incorporating narrative competency criteria into performance appraisals. Managers should also advocate for competitive compensation that recognizes narrative skills, cultivate supportive departmental cultures, and create physical environments that afford privacy for patient interactions. Additionally, nursing care delivery models and work schedules should be restructured to accommodate dedicated narrative interaction time.

At the institutional level, strategies may include establishing narrative medicine committees, streamlining administrative processes, and implementing technological solutions to reduce clerical burdens. Quality management systems should incorporate narrative competency evaluation alongside patient satisfaction metrics. Finally, collaborative efforts with nursing schools to integrate narrative medicine into curricula, as well as engagement with policymakers to promote narrative medicine integration in healthcare policy, represent important avenues for systemic change.

## 5 Conclusion

This qualitative study comprehensively explored the factors influencing nurses' medical narrative competence. The findings demonstrate that multiple interrelated factors affect nurses' narrative abilities. Personal factors include insufficient knowledge of narrative medicine, inadequate communication skills, inconsistent professional behaviors, low job satisfaction, limited self-directed learning capacity, and insufficient clinical skills. Environmental factors such as family circumstances, compensation and benefits, departmental atmosphere, and hospital humanistic culture also play significant roles in shaping nurses’ narrative abilities. Regarding relational factors, effective communication with both patients and their families is essential for narrative medicine practice. Organizational factors include limited training approaches, insufficient emphasis on narrative skill development, and inefficient healthcare processes. Enhancing nurses' medical narrative competence requires comprehensive, multi-level approaches. Future research should focus on designing and evaluating targeted intervention programs to address these identified factors.

This study has several limitations that should be acknowledged. Participant recruitment was limited to a single Grade A tertiary hospital, which may limit the generalizability of findings to nurses in different healthcare settings or regions. Additionally, while this qualitative approach provided depth of understanding regarding factors influencing nurses’ narrative abilities, it does not permit quantitative measurement or causal inference. Moreover, participants were purposively selected based on recognition for their communication or storytelling skills, which may introduce selection bias and limit transferability to the broader nursing population.

## Data Availability

The original contributions presented in the study are included in the article/Supplementary Material, further inquiries can be directed to the corresponding authors.
